# Bayesian adaptive method for estimating speed–accuracy tradeoff functions of multiple task conditions

**DOI:** 10.3758/s13428-023-02192-4

**Published:** 2023-08-07

**Authors:** Jongsoo Baek, Hae-Jeong Park

**Affiliations:** 1 Center for Systems and Translational Brain Sciences, Institute of Human Complexity and Systems Science, Yonsei University, Seoul, Republic of Korea; 2 Department of Nuclear Medicine, Department of Psychiatry, Yonsei University College of Medicine, Seoul, Republic of Korea; 3 Graduate School of Medical Science, Brain Korea 21 Project, Yonsei University College of Medicine, 50-1 Yonsei-ro, Sinchon-dong, Seodaemun-gu, Seoul, 03722 Republic of Korea; 4 Department of Cognitive Science, Yonsei University College of Medicine, 50-1 Yonsei-ro, Sinchon-dong, Seodaemun-gu, Seoul, 03722 Republic of Korea

**Keywords:** Speed-accuracy tradeoff, Adaptive procedure, Bayesian estimation, Multiple task conditions, Flanker task

## Abstract

The speed–accuracy tradeoff (SAT) often makes psychophysical data difficult to interpret. Accordingly, the SAT experimental procedure and model were proposed for an integrated account of the speed and accuracy of responses. However, the extensive data collection for a SAT experiment has blocked its popularity. For a quick estimation of SAT function (SATf), we previously developed a Bayesian adaptive SAT method, including an online stimulus selection strategy. By simulations, the method was proved efficient with high accuracy and precision with minimal trials, adequate for practically applying a single condition task. However, it calls for extensions to more general designs with multiple conditions and should be revised to achieve improved estimation performance. It also demands real experimental validation with human participants. In the current study, we suggested an improved method to measure SATfs for multiple task conditions concurrently and to enhance robustness in general designs. The performance was evaluated with simulation studies and a psychophysical experiment using a flanker task. Simulation results revealed that the proposed method with the adaptive stimulus selection strategy efficiently estimated multiple SATfs and improved performance even for cases with an extreme parameter value. In the psychophysical experiment, SATfs estimated by minimal adaptive trials (1/8 of conventional trials) showed high agreement with those by conventional trials required for reliably estimating multiple SATfs. These results indicate that the Bayesian adaptive SAT method is reliable and efficient in estimating SATfs in most experimental settings and may apply to SATf estimation in general behavioral research designs.

## Introduction

The most popular measurements in psychophysical experiments are the speed and accuracy of participants’ responses to stimuli. Since there could be a tradeoff between both measures, researchers often encounter difficulty in interpreting data. For example, suppose a participant responds to a stimulus quickly with lower accuracy in one condition but slowly with higher accuracy in another condition. In that case, it is not easy to decide under which condition the participant’s behavioral performance is superior. To solve this problem, researchers have developed an experimental paradigm and a mathematical model for the speed–accuracy tradeoff (SAT) of behavioral responses. The SAT paradigm is designed to measure the probability of correct responses (*pc*) at a wide range of response times (RT). There are several experimental manipulations for the SAT paradigm including verbal instruction, payoffs, deadlines, and response-signal to participants (see Heitz, 2014 for review). Out of these manipulations, the response-signal approach has been widely used because it can explicitly control participants’ RT. For example, in a SAT experiment with the response-signal approach (Reed, 1973), participants can respond to stimuli only when a response signal (e.g., beep tone) is being given for a short duration (e.g., 0.2s). The time delay between the stimulus onset and the response signal (stimulus-onset asynchrony; SOA) varies either trial-by-trial or block-by-block. Results are often analyzed as conjoint measures of accuracy and speed of responses. In a two-alternative forced-choice task, *pc* is modeled as a SAT function (SATf), ${\Psi }_{\theta }$, a function of RT, as follows:

1 $${\Psi }_{\theta\;=\;\left\{\lambda ,\gamma ,\delta \right\}}(x)=\left\{\begin{array}{c}\lambda \left(1-{{\text{e}}{\text{xp}}}^{-\gamma \left(x-\delta \right)}\right)+0.5, \;\text{if } x>\delta \\ 0.5, \;\text{if } x\le \delta \end{array}\right.$$

where *λ* is the asymptotic performance, *γ* is the rate parameter for the change of *pc*, *δ* is the time point where *pc* starts to increase above the chance performance (i.e., *pc* = 0.5), and *x* is RT (Wickelgren, 1977). Traditionally, SOA experiments were conducted with the method of constant stimuli (MCS). In this method, researchers predetermine a set of SOAs and repeatedly present stimuli in random order of SOA blocks. For a precise SATf estimation, a sufficient number of trials are required in each SOA block. Because of the demanding data collection for MCS (e.g., 6–8 SOA levels × 100 trials), the SAT experimental paradigm has not been popular in psychological laboratories, even though measuring SATf provides rich information about internal cognitive processes.

To make data acquisition efficient in SAT experiments, we recently developed an adaptive SAT method for psychophysical experiments (Baek & Park, 2021). The method estimates SATf rapidly with reasonable precision and accuracy by applying (1) the computational model of SATf, (2) Bayesian inference for online parameter estimation, and (3) information theory for optimal stimulus selection to the psychophysical protocol of SAT experiments (Baek et al., 2016; Hou et al., 2018; Kim et al., 2014; King-Smith et al., 1994; Kontsevich & Tyler, 1999; Kujala & Lukka, 2006; Leek, 2001). In contrast to a conventional SATf estimation by evaluating the accuracy of long block-based task trials for a set of SOAs, the proposed method is based on a Bayesian SATf estimation using trial-by-trial correctness and response time, which allows flexible SAT task design and adaptive SAT experiment by choosing the optimal SOA with maximal information gain.

By optimizing the SAT experiment, the adaptive method can efficiently estimate the parameters of SATf: our simulation results demonstrated that 50–150 adaptive trials were sufficient for accurate and precise estimation. It was 2–6 times more efficient than the conventional SAT experiment with MCS.

Although the original adaptive SAT method has the advantage of short testing time, it calls for extensions to more general designs and should be revised to achieve improved estimation performance.

First, the original adaptive SAT method is optimized for measuring a single SATf at a time but is not designed to estimate multiple SATfs concurrently. Choosing SOA for each condition to estimate condition-specific SATf would be a plausible way in some SAT task designs. However, we followed the response-signal approach for SAT task design. Most signal-based SAT task designs need to inform participants of approximate SOA as a cue for participants to prepare for each block. In this task design, the block's early trials will give the participants a hint of the SOA after that. As conditions should be mixed in a block, the method chooses one SOA regardless of conditions for each block to inform participants of the ongoing SOA level. Therefore, the new algorithm should determine the next SOA, considering the information gains of the different SATfs of both conditions.

The second problem of the original method is its vulnerability for estimating a high upper asymptote of the SATf. Because the SATf measures *pc*s in a wide range of RT, *pc*s could range from low (i.e., the chance level) to nearly perfect performance. When the task is relatively easy, a participant’s *pc* can be close to the theoretical limits (i.e., 100% correct responses) at a sufficiently large SOA, leading to a bias in the parameter estimation. This systematic bias should be resolved for the method to be robust in all possible experimental tasks or participants.

Finally, the previous study relied on simulations to test the validity of the proposed method and thus called for real experimental validation of the proposed method with human participants.

In the current study, we suggested a novel method to compensate for such limitations and to enhance robustness and applicability in measuring SATf in general designs. The robustness of the proposed method was evaluated with simulation studies and a psychophysical experiment using a flanker experiment.

## Algorithm of the Bayesian adaptive SAT method for multiple conditions

The adaptive SATf procedure (Baek & Park, 2021) can briefly be summarized as follows. Starting with a prior probability distribution over parameters of the function (i.e., *λ*, *γ,* and *δ*), the method selects the most informative SOA: for each SOA, it computes the expected information gain or entropy of the probability distribution when stimuli are presented with the SOA. Stimuli are presented with the selected SOA in the next block, and participants respond to the stimuli. After the Bayesian update of the probability distribution based on the participant’s responses at each trial, parameter estimates can be calculated from the expected values of the probability distribution (Kontsevich & Tyler, 1999; Lesmes et al., 2006; Watson & Pelli, 1983). The method is iterated until the predetermined number of blocks. This section describes the mathematical formulation of the initial method (Baek & Park, 2021), followed by an extension to tasks with multiple conditions.

1. Exploration of SATf, ${\Psi }_{{\theta }_{c}}$ for a condition $c$ (Eq. 1) in an individual, by iteratively estimating a set of parameters ${\theta }_{c}=\{{\lambda }_{c},{\gamma }_{c},{\delta }_{c}\}$, begins with assigning the three-dimensional prior probability distribution, ${p}_{0}({\theta }_{c})$.
2. After each trial, the method updates the posterior probability distribution considering the participant’s response with the following steps:
  a A stimulus is presented with the SOA of *T*.
  b The method records the participant’s binarized response ${r}_{x}$ (either 1 for correct or 0 for incorrect) with an RT, *x*.
  c The likelihood of getting a response ${r}_{x}$ for RT *x* given ${\theta }_{c}$, $p\left({r}_{x}|{\theta }_{c}\right)={\Psi }_{{\theta }_{c}}(x)$, and is computed for all parameter space of ${\theta }_{c}$ using SATf, ${\Psi }_{{\theta }_{c}}(x)$ to reduce calculation cost.
  d By the Bayesian theorem, the prior distribution at the *t*-th trial, ${p}_{t}\left({\theta }_{c}\right)$, is updated to the posterior distribution, ${p}_{t}({\theta }_{c}|{r}_{x})$, with the newly observed response ${r}_{x}$ with RT *x*:

2 $${p}_{t}({\theta }_{c}|{r}_{x})=\frac{{p}_{t}({\theta }_{c})p({r}_{x}|{\theta }_{c})}{{p}_{t}({r}_{x})}$$

The probability of getting a response ${r}_{x}$ with RT *x,* ${p}_{t}({r}_{x})$ at the *t*-th trial, is evaluated by weighting the empirical response probability with the prior:

3 $${p}_{t}({r}_{x})=\sum_{{\theta }_{c}}{p}_{t}({\theta }_{c})p({r}_{x}|{\theta }_{c})$$

e. The posterior distribution at the *t*-th trial is used as the prior distribution at the next trial,

4 $${p}_{t+1}({\theta }_{c})={p}_{t}({\theta }_{c}|{r}_{x})$$

3. After each block, composed of multiple trials of all conditions, the method selects the most informative SOA level *T* for the next block. Here, we chose the SOA level with the maximum expected information gain among all possible SOAs. In this step, an SOA should be determined considering all the conditions in the block, with the probability density estimated independently between conditions. For simplicity, we exampled a task with two-conditions without loss of generality. We specified the above ${\theta }_{c}$ to ${\theta }_{1}$ for condition 1 and ${\theta }_{2}$ for condition 2. We assumed the ${\theta }_{1}$ and ${\theta }_{2}$ for SATf are independent and the responses ${r}_{\widehat{x}}^{\left(1\right)},{r}_{\widehat{x}}^{\left(2\right)}$ for condition 1 and condition 2 with a given SOA level, denoted by *T*, with an expected RT, denoted by $\widehat{x}$, are also independent. We assume that the expected RT($\widehat{x}$), for a given SOA (*T*), is the midpoint of a 0.2-sec time window for response (i.e., $\widehat{x}=T+0.1$). In this block-based presentation, we denote ${r}_{\widehat{x}}^{\left(1\right)},{r}_{\widehat{x}}^{\left(2\right)}$ for specific values of the random variables ${R}_{\widehat{x}}^{\left(1\right)},{R}_{\widehat{x}}^{\left(2\right)}$, which represent the numbers of correct responses (*r*) for conditions in a block of *n* trials placed at $\widehat{x}$. Note that uppercase letters represent random variables and lowercase letters represent specific values. The adaptive SAT procedure for multiple task conditions proposed in this study can be summarized below.
  a For a block including *n*_*c*_ trials for a condition *c*, we denote $p\left({r}_{\widehat{x}}^{\left(c\right)}|{\theta }_{c}\right)$ as the probability of getting the number of correct responses ${r}_{\widehat{x}}^{\left(c\right)}$ for a given expected RT $\widehat{x}$ and parameter *θ*_*c*_.

5 $$p\left({r}_{\widehat{x}}^{\left(c\right)}|{\theta }_{c}\right)\triangleq p\left({{R}_{\widehat{x}}^{\left(c\right)}=r}_{\widehat{x}}^{\left(c\right)}|\widehat{x},{{n}_{c},\theta }_{c}\right)=\left(\genfrac{}{}{0pt}{}{{n}_{c}}{{r}_{\widehat{x}}^{\left(c\right)}}\right){{\Psi }_{{\theta }_{c}}\left({r}_{\widehat{x}}^{\left(c\right)}\right)}^{{r}_{\widehat{x}}^{\left(c\right)}}{\left(1-{\Psi }_{{\theta }_{c}}\left({r}_{\widehat{x}}^{\left(c\right)}\right)\right)}^{{n}_{c}-{r}_{\widehat{x}}^{\left(c\right)}}$$

The probability of independently getting ${r}_{\widehat{x}}^{\left(1\right)},{r}_{\widehat{x}}^{\left(2\right)}$ from each condition for a given level of $\widehat{x}$ with independent SATf parameters ${\theta }_{1},{\theta }_{2}$ can be derived by multiplying each condition’s $p\left({r}_{\widehat{x}}^{\left(c\right)}|{\theta }_{c}\right)$ as below.

6 $$p\left({r}_{\widehat{x}}^{\left(1\right)},{r}_{\widehat{x}}^{\left(2\right)}|{\theta }_{1},{\theta }_{2}\right)\triangleq p\left({{R}_{\widehat{x}}^{\left(1\right)}=r}_{\widehat{x}}^{\left(1\right)},{{R}_{\widehat{x}}^{\left(2\right)}=r}_{\widehat{x}}^{\left(2\right)}|\widehat{x},{{n}_{1},\theta }_{1},{{n}_{2},\theta }_{2}\right)=\prod_{c=1}^{2}p\left({r}_{\widehat{x}}^{\left(c\right)}|{\theta }_{c}\right)$$

b. The method computes entropy for the variables for the correct response numbers ${R}_{\widehat{x}}=\{{R}_{\widehat{x}}^{\left(1\right)},{R}_{\widehat{x}}^{\left(2\right)}\}$ for two conditions in an upcoming (*b+*1)-th block, under a set of parameters $\theta =\{{\theta }_{1},{\theta }_{2}\}$ for the two conditions updated after *b*-th block data acquisition with a given *SOA* according to Eq. (4), as a function of $\widehat{x}$*.*
  7 $${H}_{b+1}\left({R}_{\widehat{x}}|\theta \right)\triangleq -\sum_{{r}_{\widehat{x}}^{\left(1\right)}\in \left\{\mathrm{0,1},\cdots ,{n}_{1}\right\}}\sum_{{r}_{\widehat{x}}^{\left(2\right)}\in \left\{\mathrm{0,1},\cdots ,{n}_{2}\right\}}{p}_{b+1}\left({r}_{\widehat{x}}^{\left(1\right)},{r}_{\widehat{x}}^{\left(2\right)}|{\theta }_{1},{\theta }_{2}\right)\mathrm{log}{p}_{b+1}\left({r}_{\widehat{x}}^{\left(1\right)},{r}_{\widehat{x}}^{\left(2\right)}|{\theta }_{1},{\theta }_{2}\right)$$

The expected conditional entropy is derived as

8 $${H}_{b+1}\left({R}_{\widehat{x}}|\Theta \right)\triangleq \sum_{{\theta }_{1}}\sum_{{\theta }_{2}}{p}_{b+1}({\theta }_{1},{\theta }_{2})H({R}_{\widehat{x}}|{\theta }_{1},{\theta }_{2})$$

In Eqs. (7) and (8), ${p}_{b+1}$ indicates probability function evaluated for the (*b+*1)-th block using the parameters $\theta$ estimated before the block. For brevity, we skipped notation *b* or *b*+1 for ${r}_{\widehat{x}}^{\left(1\right)},{r}_{\widehat{x}}^{\left(2\right)},{\theta }_{1},{\theta }_{2}$.

The joint entropy of two independent variables $X \text{ and } Y$ is the sum of entropy of each variable, i.e., $H\left(X,Y\right)=H\left(X\right)+X(Y)$. If $X$ is conditionally independent of ${Y}_{2}$ given ${Y}_{1}$, we have $H\left(X|{Y}_{1},{Y}_{2}\right)=H\left(X|{Y}_{1}\right)$. From these relationships, we can derive the following equation with a sum of expected conditional entropy of each independent condition.

9 $${H}_{b+1}\left({R}_{\widehat{x}}|\Theta \right)={H}_{b+1}\left({R}_{\widehat{x}}^{\left(1\right)}|\Theta \right)+{H}_{b+1}\left({R}_{\widehat{x}}^{\left(2\right)}|\Theta \right)={H}_{b+1}\left({R}_{\widehat{x}}^{\left(1\right)}|{\Theta }_{1}\right)+{H}_{b+1}\left({R}_{\widehat{x}}^{\left(2\right)}|{\Theta }_{2}\right)$$

The entropy of responses ${H}_{b+1}\left({R}_{\widehat{x}}^{\left(c\right)}\right)$ is defined as below.

10 $${H}_{b+1}\left({R}_{\widehat{x}}^{\left(c\right)}\right)\triangleq \sum_{{r}_{\widehat{x}}^{\left(c\right)}\in \left\{\mathrm{0,1},\cdots ,{n}_{c}\right\}}{p}_{b+1}\left({r}_{\widehat{x}}^{\left(c\right)}\right)\mathrm{log}{p}_{b+1}\left({r}_{\widehat{x}}^{\left(c\right)}\right)$$

11 $${p}_{b+1}\left({r}_{\widehat{x}}^{\left(c\right)}\right)={p}_{b+1}\left({R}_{\widehat{x}}^{\left(c\right)}={r}_{\widehat{x}}^{\left(c\right)}\right)=\sum_{{\theta }_{c}}{p}_{b+1}({\theta }_{c})p\left({r}_{\widehat{x}}^{\left(c\right)}|{\theta }_{c}\right)$$

As ${R}_{\widehat{x}}^{\left(1\right)}$ and ${R}_{\widehat{x}}^{\left(2\right)}$ are independent, the entropy of responses ${H}_{b+1}\left({R}_{\widehat{x}}\right)$ is derived as below (see Appendix).

12 $${H}_{b+1}\left({R}_{\widehat{x}}^{\left(1\right)},{R}_{\widehat{x}}^{\left(2\right)}\right)\triangleq \sum_{{r}_{\widehat{x}}^{\left(1\right)}\in \left\{\mathrm{0,1},\cdots ,{n}_{1}\right\}}\sum_{{r}_{\widehat{x}}^{\left(2\right)}\in \left\{\mathrm{0,1},\cdots ,{n}_{2}\right\}}{p}_{b+1}\left({r}_{\widehat{x}}^{\left(1\right)},{r}_{\widehat{x}}^{\left(2\right)}\right)\mathrm{log}{p}_{b+1}\left({r}_{\widehat{x}}^{\left(1\right)},{r}_{\widehat{x}}^{\left(2\right)}\right)={H}_{b+1}\left({R}_{\widehat{x}}^{\left(1\right)}\right)+{H}_{b+1}\left({R}_{\widehat{x}}^{\left(2\right)}\right)$$

iii. The expected information gain for the next block *b+1* is computed for each SOA level *T* with $\widehat{x}$ by evaluating mutual ﻿information:
  13 $${I}_{b+1}\left({R}_{\widehat{x}};\Theta \right)={H}_{b+1}\left({R}_{\widehat{x}}\right)-{H}_{b+1}\left({R}_{\widehat{x}}|\Theta \right)={H}_{b+1}\left({R}_{\widehat{x}}^{\left(1\right)}\right)+{H}_{b+1}\left({R}_{\widehat{x}}^{\left(2\right)}\right)-{H}_{b+1}\left({R}_{\widehat{x}}^{\left(1\right)}|\Theta \right)-{H}_{b+1}\left({R}_{\widehat{x}}^{\left(2\right)}|\Theta \right)={H}_{b+1}\left({R}_{\widehat{x}}^{\left(1\right)}\right)-{H}_{b+1}\left({R}_{\widehat{x}}^{\left(1\right)}|{\Theta }_{1}\right)+{H}_{b+1}\left({R}_{\widehat{x}}^{\left(2\right)}\right)-{H}_{b+1}\left({R}_{\widehat{x}}^{\left(2\right)}|{\Theta }_{2}\right)={I}_{b+1}\left({R}_{\widehat{x}};{\Theta }_{1}\right)+{I}_{b+1}\left({R}_{\widehat{x}};{\Theta }_{2}\right)$$

iv. The SOA level ${T}_{b+1}$ with the maximum expected information gain ${I}_{b+1}({R}_{\widehat{x}};\Theta )$ is chosen for the next block.

4. The method iterates steps 2–3 until a predefined number of blocks.
5. After the experiment is completed, the parameter of SATf is estimated by computing the mean of the marginal posterior distribution for each condition. The SATf is calculated by averaging 1000 SATfs resampled from the posterior distribution.

## Simulation 1. Concurrent measurement of multiple SATfs

**Fig. 1:**
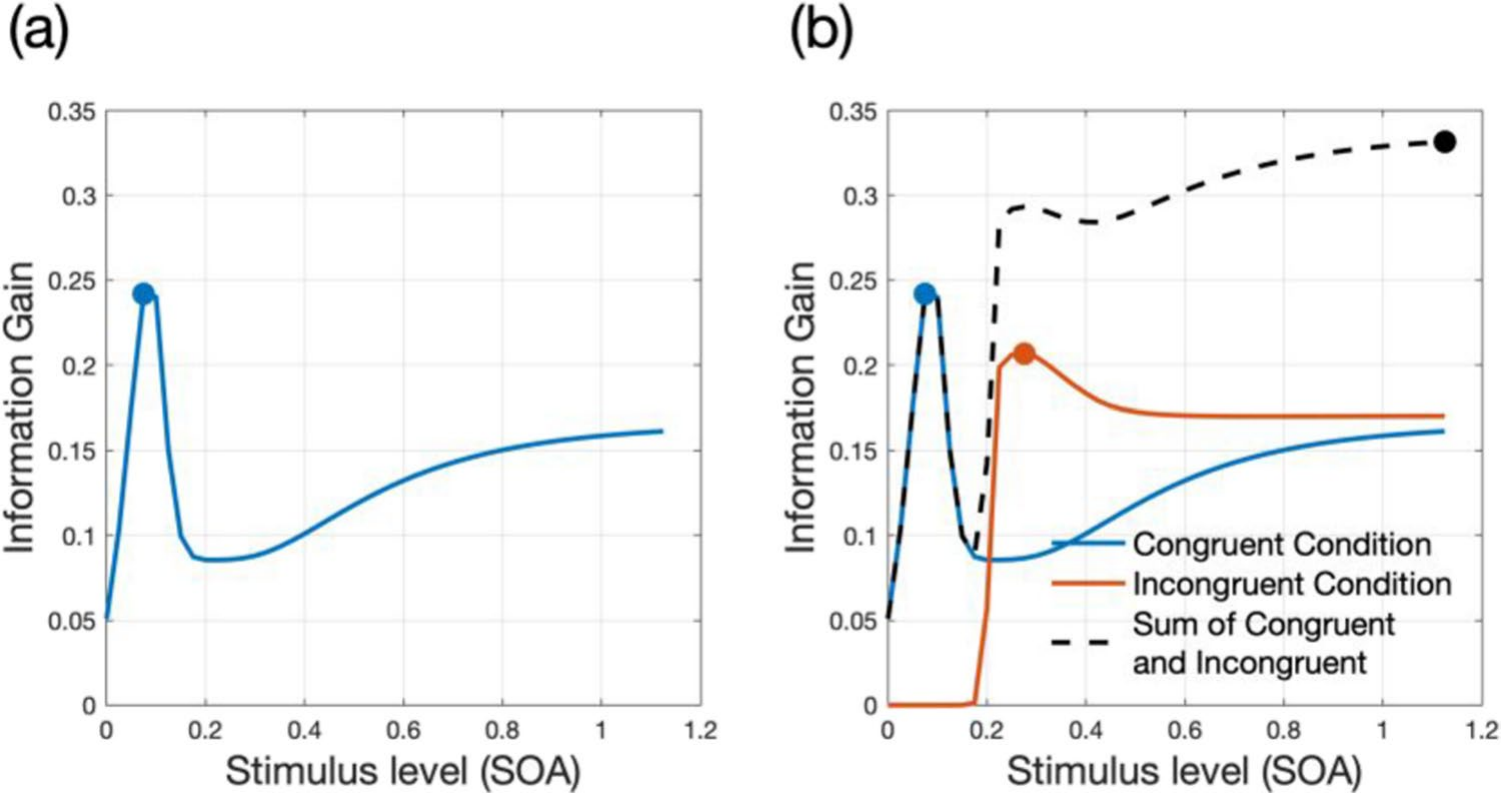
Hypothetical information gain as a function of SOA. (**a**) When a SOA block includes a single condition, there is one ‘most informative stimulus level’ (*circle*), which is the maximum in the expected information gain function (*line*). (**b**) When a SOA block includes multiple conditions, there could be multiple ‘most informative stimulus levels’ (*circles*). In this case, the most informative stimulus level could be determined by summing the expected information gain functions over all conditions (*black dashed line*)

The core of the adaptive SAT method is that it searches for the stimulus space to find which stimulus will provide more information about a participant’s SATf. By predicting the expected information gain of each stimulus level, the method determines the most informative SOA and presents stimuli with the SOA in the next block. Figure 1a illustrates a hypothetical information gain as a function of SOA, in which the expected information gain is maximum at SOA of 0.075s. The function implies that trials could provide three times more information by testing SOA of 0.075s than 0.2s. As a result, the method can estimate SATf more quickly by presenting stimuli with this SOA. Our previous study proved that the strategy of optimal stimulus selection improved the performance of the SATf estimation procedure: the method estimates SATfs more accurately, precisely, and efficiently with than without the optimal stimulus selection (Baek & Park, 2021).

Such a strategy, however, does not apply to all experimental designs. In a study with multiple conditions, i.e., a within-subject design, the SATf estimation, using a single condition-based analysis independently from other conditions, would not always be accurate and precise. This is because the “most” informative stimulus could be more than one when SATfs are estimated for two or more experimental conditions concurrently. Figure 1b depicts a situation with two information gain functions, one for each condition (e.g., congruent and incongruent conditions in a flanker task). The most informative stimulus level is different between conditions: the maximum point of information gain function is at SOA of 0.075 and 0.275 s in the congruent and incongruent conditions, respectively. If the method selects and tests SOA of 0.075 s, the response data provides more information about the participant’s SATf than any other possible SOAs for the congruent condition but almost no information for the incongruent condition. In the end, the SATf estimation would be great for the congruent condition but poor for the incongruent condition. This would be the case when the method selects the next SOA based on the information gain of the incongruent condition.

To make the method robust to the concurrent estimation of multiple SATfs, we propose a new strategy: selecting the optimal SOA based on the block information gain described in Eq. (13), which equals to the sum of information gain functions over multiple conditions. The sum of information gain from both conditions has a peak at 1.125 s. Thus, by testing SOA of 1.125 s, the method could acquire a good amount of information for both conditions and achieve the maximum information gain considering all conditions.

To evaluate the performance—accuracy, precision, and efficiency—of the adaptive SAT method with the sum-based stimulus selection for measuring SATfs in multiple conditions, we performed a simulation study. In a batch of simulations, two virtual participants ran a SAT version of the flanker task with the response-signal manipulation. The method estimated participants’ SATfs with different SOA selection strategies: (1) Strategy 1 for the SOA selection based on information gain of congruent condition, (2) Strategy 2 based on incongruent condition, and (3) Strategy 3 based on the sum of them derived in Eq. (13).

## Methods

In the current experimental setting, two conditions (i.e., congruent and incongruent) were interleaved in each block, and participants had different SATfs for conditions. We simulated three virtual participants with a range of SAT parameters for incongruent conditions. The difference between δ of true SATfs was relatively small in participants 1 and 3 but large in participant 2. The true γ’s were the same for both conditions in participants 1 and 2 but different in participant 3 (See Table 1 for the parameters of participants).

**Table 1:** SAT parameters of virtual participants in simulation 1

	Congruent			Incongruent		
	λ	γ	δ	λ	γ	δ
Participant 1	0.45	25	**0.20**	0.45	25	**0.30**
Participant 2	0.45	25	**0.20**	0.45	25	**0.45**
Participant 3	0.45	**25**	**0.20**	0.45	**5**	**0.45**

RT and correctness of responses were simulated for SOAs between 0 and 1.2 s. In each trial, RT, *x*, was generated randomly from an ex-Gaussian distribution (*μ* = .3, *σ* = .06, *τ* = .08), with restrictions of the selected SOA and the time window for the response of 0.2 s (i.e., SOA < *x* < SOA + 0.2 s). We divided a single ex-Gaussian distribution function into multiple partitions according to SOAs and normalized each partition’s distribution to make the sum of the partition’s distribution one. We then generated RT samples according to each partition’s normalized distribution for each SOA. The expected probability of correct, *pc*(*x*) was calculated for RT, *x* using Eq. (1). The correctness of the response was simulated by a random number *r* generated from a uniform distribution in the range between 0 and 1. The response was marked as correct if *r* < *pc*(*x*) and incorrect otherwise. Each run consisted of 256 trials (= 16 trials × 8 SOA blocks) for each congruency. Simulated runs were iterated 1000 times for each participant. The prior distribution was uniform over 21 linearly spaced *λ* values (from .4 to .5 or .55), 30 linearly spaced *γ* values (from 1 to 30), and 25 linearly spaced *δ* values (from .02 to .5). Possible SOAs ranged from 0 to 1.2 s in 0.025-s steps (i.e., 49 linearly spaced samples).

## Results

We assessed the accuracy of estimated SATfs with the mean absolute error (MAE) between true and estimated SATfs and precision by the half-width of 68.2% credible interval (HWCI) of the posterior distribution averaged over 49 SOAs. The MAE of the estimated SATf can be calculated as:

14 $${MAE}_{i}=\frac{\sum_{k=1}^{K}\left|{\sum }_{j=1}^{J}{(pc}_{ij}^{k}-{pc}_{true}^{k})\right|}{J\times K}$$

where ${pc}_{ij}^{k}$ is the estimated *pc* for *k*-th SOA (out of 49 linearly spaced SOAs between 0 to 1.2 s) at the *i*-th trial of the *j*-th run and ${pc}_{true}^{k}$ is the true *pc* for the *k*-th SOA. Results are summarized in Tables 2 and 3.

**Table 2:** Accuracy of the adaptive SAT methods in Simulation 1

	MAE						Trials for MAE = 0.010					
	Congruent			Incongruent			Congruent			Incongruent		
	P1	P2	P3	P1	P2	P3	P1	P2	P3	P1	P2	P3
Strategy 1	0.006	0.006	0.006	**0.020**	**0.038**	**0.034**	80	82	81	**> 256**	**> 256**	**>2 56**
Strategy 2	**0.014**	**0.023**	**0.023**	0.004	0.006	0.006	**> 256**	**> 256**	**> 256**	66	89	129
Strategy 3	0.006	0.007	0.006	0.006	0.008	0.010	98	128	120	84	144	242

Low accuracy (i.e., MAE > 0.010, and trials for MAE = 0.010 are greater than 256) is highlighted in bold text. P1, Participant 1; P2, Participant 2; P3, Participant 3

**Table 3:** Precision of the adaptive SAT methods in Simulation 1

	HWCI						Trials for HWCI = 0.020					
	Congruent			Incongruent			Congruent			Incongruent		
	P1	P2	P3	P1	P2	P3	P1	P2	P3	P1	P2	P3
Strategy 1	**0.021**	**0.021**	**0.021**	**0.030**	**0.040**	**0.040**	**> 256**	**> 256**	**> 256**	**> 256**	**> 256**	**> 256**
Strategy 2	**0.022**	**0.027**	**0.027**	0.017	0.016	0.019	**> 256**	**> 256**	**> 256**	166	138	215
Strategy 3	0.018	0.017	0.017	0.019	0.020	**0.026**	175	171	158	193	233	**> 256**

Low precision (i.e., HWCI > 0.020, and trials for HWCI = 0.020 are greater than 256) is highlighted in bold texts. P1, Participant 1; P2, Participant 2; P3, Participant 3

We empirically determined the cut-off values between accurate and precise or inaccurate and imprecise estimation, referring to classic SAT studies. A single SATf estimation required 1920 MCS trials in one study (Reed, 1973) and 4060 trials in another (Reed, 1976). Our previous study (Baek & Park, 2021) showed approximately MAE = .013 with 1920 MCS trials. MAE at 4060 MCS trials is unknown but expected to be smaller than .013. We also calculated the standard error of MCS estimation (averaged across all SOAs and observers we simulated). It was .0240 after 1920 MCS trials and .0165 after 4060 MCS trials. Thus, we regarded MAE = .01 and HWCI = .02 as appropriate criteria for accurate and precise estimation.

**Fig. 2:**
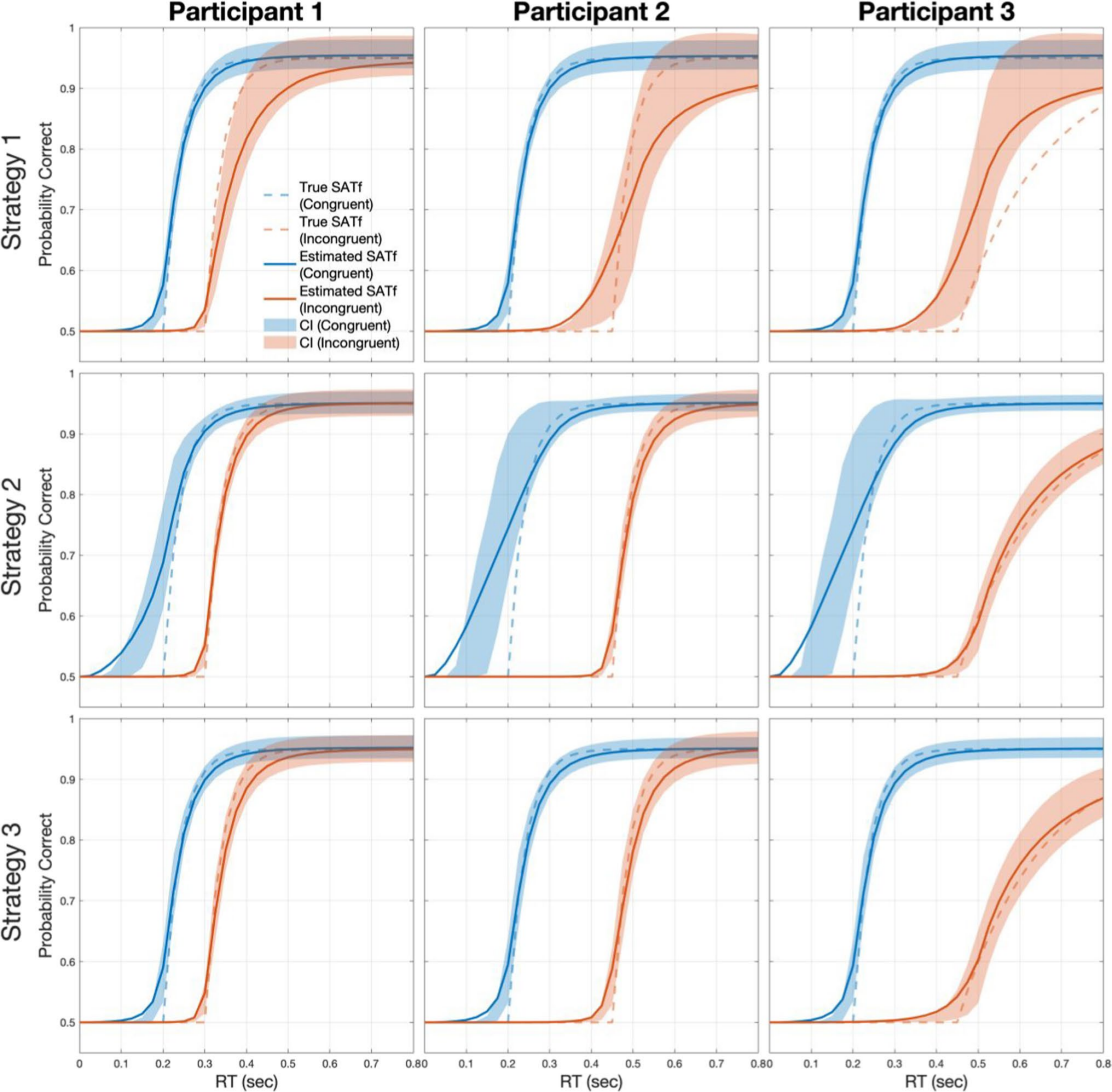
SATfs estimated with 256 adaptive trials using different stimulus selection strategies. With Strategy 1 (*upper panels*), estimation shows good accuracy (little difference between dashed and solid lines) and precision (*narrow shaded areas*) for the congruent condition but not for the incongruent condition. It is vice versa with Strategy 2 (*middle panels*). Estimation with Strategy 3 shows good accuracy and precision for both conditions (*middle panels*)

With Strategy 1 (upper panels in Fig. 2), the method estimated SATfs accurately and precisely in the congruent condition (MAE = 0.006 and HWCI = 0.021 for all participants). On the contrary, the estimation was not accurate nor precise for any participant in the incongruent condition (MAE = 0.020, 0.038, and 0.034; HWCI = 0.030, 0.040, and 0.040 for participants 1, 2, and 3, respectively). This was also the case with Strategy 2 (middle panels in Fig. 2). The estimated SATf was neither accurate nor precise in the congruent condition (MAE = 0. 014, 0.023, and 0.023; HWCI = 0.022, 0.027, and 0.027 for participants 1, 2, and 3), although it was in the incongruent condition (MAE = 0. 004, 0. 006 and, 0. 006; HWCI = 0.017, 0.016, and 0.019 for participants 1, 2 and 3). With Strategy 1 and 2, the method well estimated SATf for the targeted condition, which the stimulus selection algorithm was optimized for, but not for the untargeted condition. However, the estimation performance became robust for both congruent and incongruent conditions when the method adopted Strategy 3 (MAE < 0.008 and HWCI < 0.020 for all participants × congruency conditions except for the incongruent condition in participant 3; lower panels in Fig. 2). The accuracy and precision with Strategy 3 were slightly higher than Strategy 2 for the incongruent condition but were greatly improved for the congruent condition. For participant 3 with an extreme parameter, the estimation was somewhat imprecise in the incongruent condition (HWCI = .026). In determining the optimal SOA for participant 3, the algorithm had poor information gain as the highly informative SOA zone for estimating SATf in the congruent condition does not provide information on the incongruent condition and vice versa. The lower precision particularly in the incongruent condition in patient 3 might be caused by highly deviated parameters from the prior range assigned for the general population. Thus, the maximum SOA set for the general population could be insufficient to accurately and precisely estimate the right tail of the SATf for participant 3. To solve this problem, we first need to extend the maximum range of SOA more than the conventional one. Even if we extend the SOA range, the less overlap between the two conditions hinders taking high advantage of the adaptive method because the optimal SOA with the maximal information gain for multiple conditions is not clear due to the disjoint of the SATfs for both conditions.

**Fig. 3:**
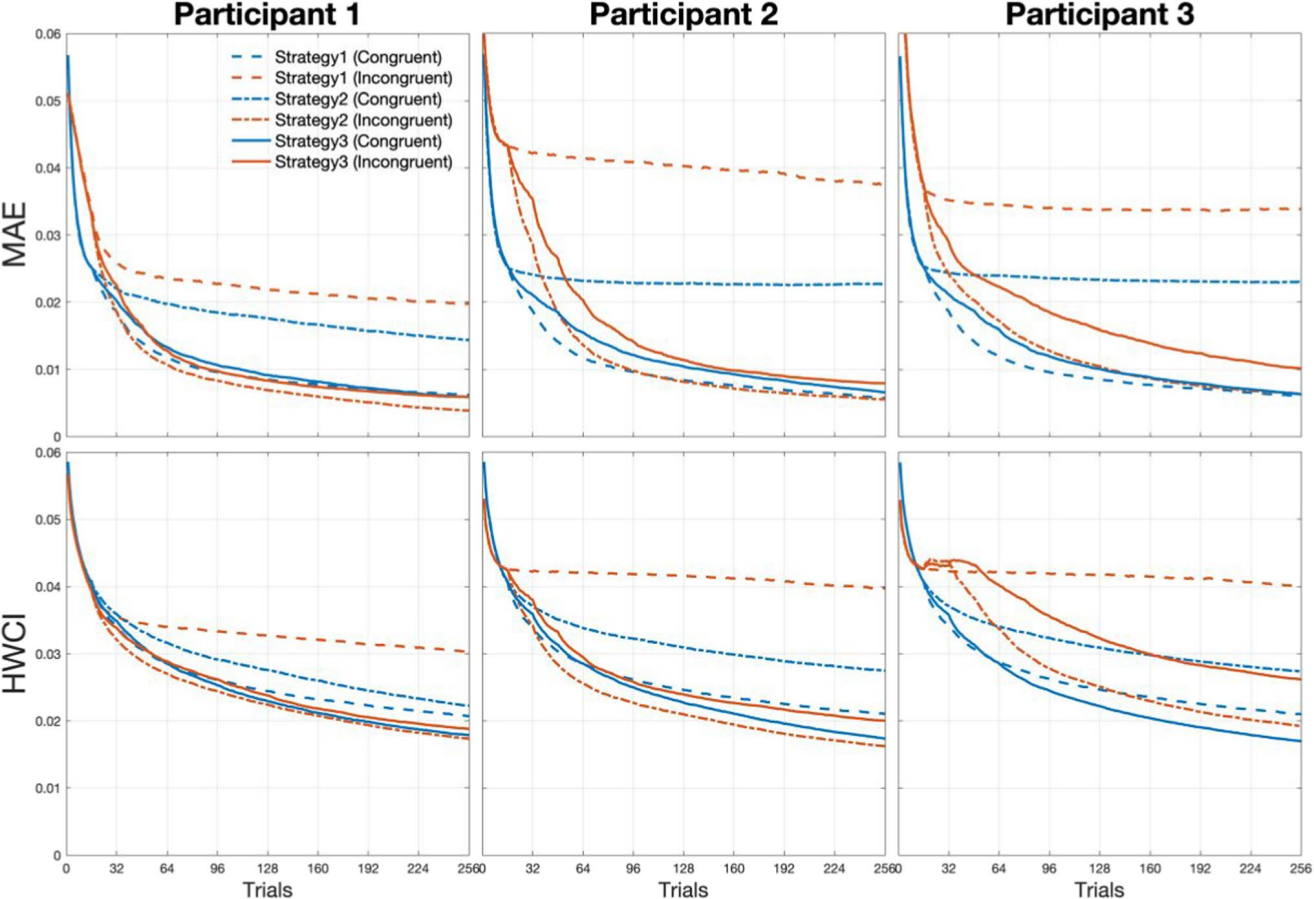
Accuracy (MAE; *upper panels*) and precision (HWCI; *lower panels*) of the SATf estimation using different stimulus selection strategies as a function of completed trial numbers. Estimation with Strategy 1 (*dashed lines*) and 2 (*dotted lines*) always shows poorer accuracy and precision for the untargeted condition. Estimation with Strategy 3 (*solid lines*) shows good performance in estimating SATfs of both conditions

We also evaluated the efficiency of the SATf estimation method. The efficiency can be defined as a trial number required to reach a certain level of accuracy and precision (e.g., MAE < .01 and HWCI < .02). Accuracy and precision of the estimated SATf were plotted against the completed trial numbers in Fig. 3. With Strategies 1 and 2, the method always exhibited poorer estimation performance for the untargeted condition. With Strategy 2, accuracy and precision in the congruent condition started to saturate at relatively higher MAE and HWCI after several trials (i.e., 16–32) and did not improve significantly afterward. The method with Strategy 1 showed less accurate and precise estimation for the incongruent condition over all trials of the simulated experiment. In contrast, Strategy 3 made the method to measure SATfs of both conditions accurately and precisely for all participants. The method required only 144 trials to reach the accuracy of MAE = 0.01 and 256 trials to achieve the precision of HWCI = 0.02, except for the incongruent condition of participant 3 (see Tables 2 and 3). The simulation indicated that the stimulus selection based on a single condition caused the problem of low accuracy and precision in estimating SATfs for an untargeted condition and that such a problem can be minimized by adopting the stimulus selection strategy based on the sum of the information gain of all conditions.

## Simulation 2. Avoiding the underestimation of extreme values

**Fig. 4:**
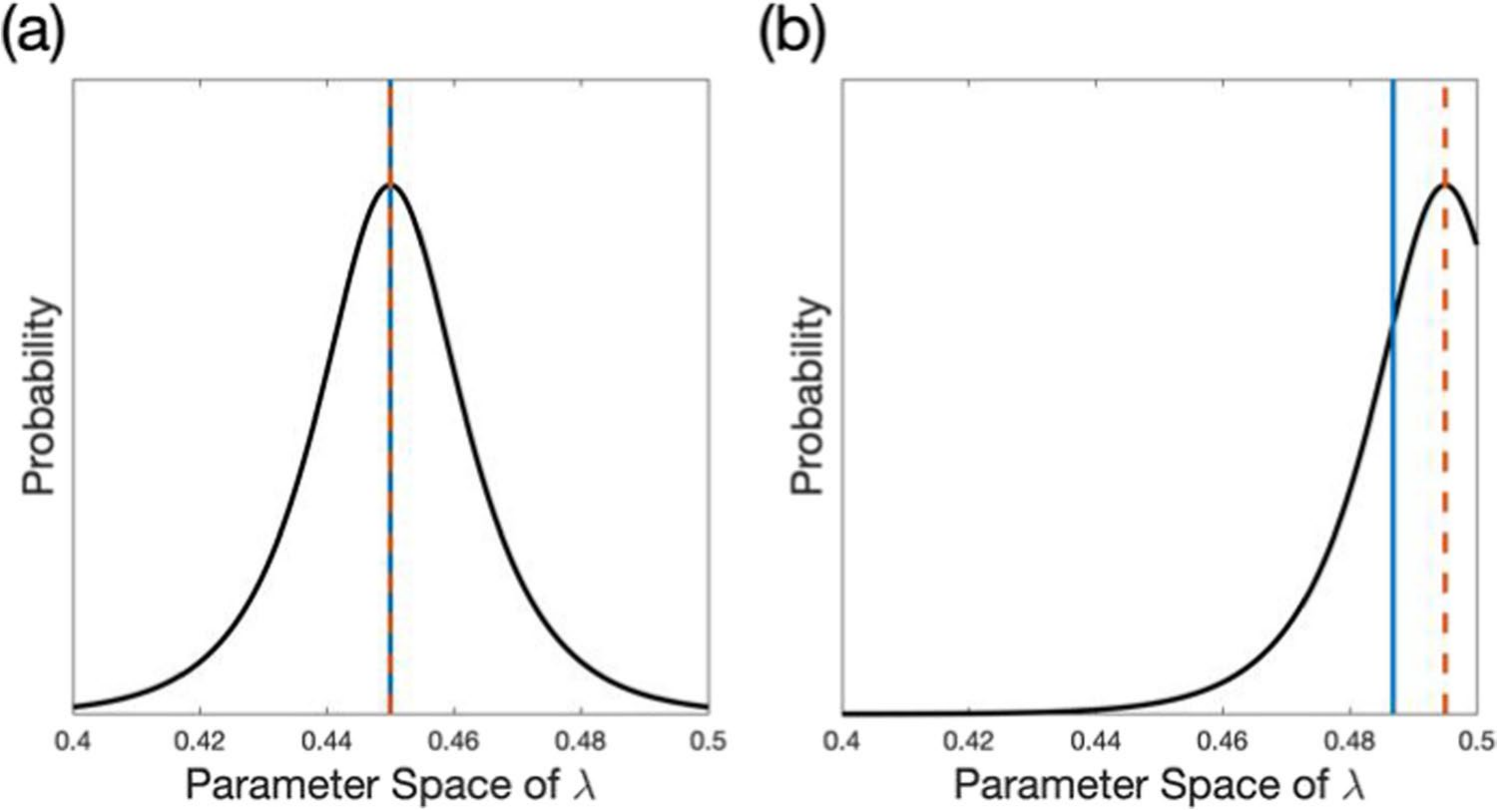
Hypothetical marginal distribution of posterior probability for *λ*. (**a**) When the distribution is located at the center of parameter space, the estimate computed by the mean of the posterior distribution (*blue*) is well matched to the mode (*λ* with the highest probability; *red*). (**b**) When the probability density is highest around the border of parameter space, the mean of the distribution is lower than the mode, resulting in an underestimation of the parameter value

In the adaptive SAT method, each parameter is estimated by calculating the mean of the marginal posterior distribution. Although this approach has been known as accurate and precise in most cases (Baek et al., 2016; Baek & Park, 2021; Kontsevich & Tyler, 1999; Watson & Pelli, 1983), it poses a systemic bias occasionally. When the distribution has a peak at the center of the defined *λ* space (i.e., 0.4–0.5; Fig. 4a), the mean of the distribution is well matched to the most likely parameter value (mean of 0.45 vs. mode of 0.45), resulting in the accurate estimation for *λ*. In an easy task or a participant with lower lapse, *pc* could be almost perfect for the stimuli with a sufficient SOA so that the true *λ* is close to 0.5. When the probability density is highest around the border of parameter space (Fig. 4b), *λ* could be underestimated since the mean of the distribution is lower than the most likely parameter value (mean of 0.487 vs. mode of 0.495). That is, the estimation with the Bayesian adaptive SAT method could be biased toward the center of the parameter space.

To improve the robustness of the method for estimating a SATf with extreme parameter values, we suggest a modified method: setting up the parameter space of *λ* broader beyond the theoretical limit (i.e., 0.5) and then truncating the estimated parameter if it is greater than the limit. To investigate whether this modification could correct the underestimation of *λ*, we simulated two participants with and without the adjustment of parameter space.

## Methods

The methods were the same as in Simulation 1. The asymptote parameter, *λ*, was in the middle of the parameter space for participant 1 (*λ* = 0.45), but close to the upper bound of the space for participant 2 (*λ* = 0.495). The parameters of simulated participants are listed in Table 4. We estimated their SATfs using the adaptive SAT method with and without adjusting *λ* space. The *λ* space ranged from 0.4 to 0.5 for the method without adjustment and from 0.4 to 0.55 for the method without adjustment.

**Table 4:** SAT parameters of virtual participants in simulation 2

	Congruent			Incongruent		
	*λ*	*γ*	*δ*	*λ*	*γ*	*δ*
Participant 1	**0.45**	25	0.20	**0.45**	25	0.30
Participant 2	**0.495**	25	0.20	**0.495**	25	0.30

## Results

**Fig. 5:**
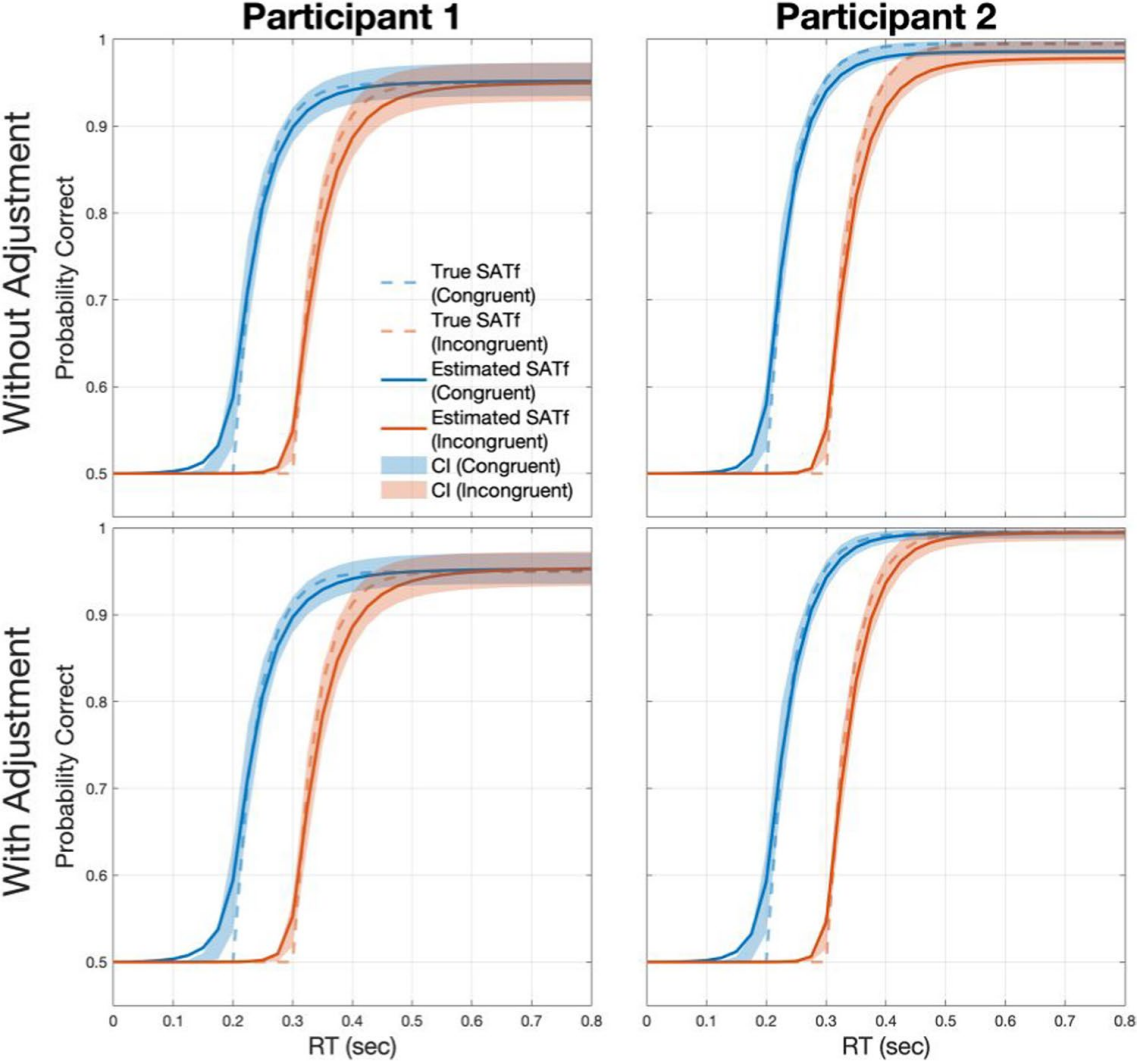
SATfs estimated with 256 adaptive trials without (*upper panels*) and with the adjustment (*lower panels*) of parameter space. For participant 1, accuracy and precision are comparable between the procedure with and without the adjustment. For participant 2, the method shows better accuracy and precision with the adjustment than without adjustment

The method with adjustment demonstrated improved accuracy and precision for the high-performing participant than without adjustment (Fig. 5, Tables 5 and 6). For participant 1, whose *λ* is in the middle of parameter space, SATfs estimated by both methods with 256 trials were close to the true SATfs. Precision was comparable between the method with and without the adjustment. For the method without the adjustment, MAE between true and estimated SATfs was 0.006 for both congruencies and HWCI was 0.018 and 0.019 for the congruent and incongruent conditions, respectively. Similarly, for the method with the adjustment, MAE was 0.007 for both congruencies and HWCI was 0.017 and 0.018 for the congruent and incongruent conditions, respectively. For participant 2, whose *λ* was close to the upper bound of *λ* space (i.e., *λ* = 0.495), the estimation performance was better for the method with the adjustment than without it. The method shows a large error, especially for the longer SOAs, which reflects a significant bias in *λ*, in the incongruent condition without the adjustment (MAE = 0.010 and 0.016 for the congruent and incongruent conditions, respectively), but significantly improved accuracy with the adjustment (MAE = 0. 005 and 0.004 for the congruent and incongruent conditions, respectively). The precision of the method was good even without the adjustment (HWCI = 0.009 and 0.013 for the congruent and incongruent conditions, respectively), but further improved with the adjustment (HWCI = 0.008 for both congruencies). These results illustrated that adjusting *λ* space resulted in a more accurate and precise estimation of overall SATf.

**Table 5:** Accuracy of the adaptive SAT methods in Simulation 2

	MAE				Trials for MAE = 0.010			
	Congruent		Incongruent		Congruent		Incongruent	
	P1	P2	P1	P2	P1	P2	P1	P2
without adjustment	0.006	0.010	0.006	**0.016**	92	247	83	**> 256**
with adjustment	0.007	0.005	0.007	0.004	151	94	156	92

Low accuracy (i.e., MAE > 0.010, and trials for MAE = 0.010 are greater than 256) is highlighted in bold texts

**Table 6:** Precision of the adaptive SAT methods in Simulation 2

	HWCI				Trials for HWCI = 0.020			
	Congruent		Incongruent		Congruent		Incongruent	
	P1	P2	P1	P2	P1	P2	P1	P2
without adjustment	0.018	0.009	0.019	0.013	175	91	191	133
with adjustment	0.017	0.008	0.018	0.008	164	53	130	60

**Fig. 6:**
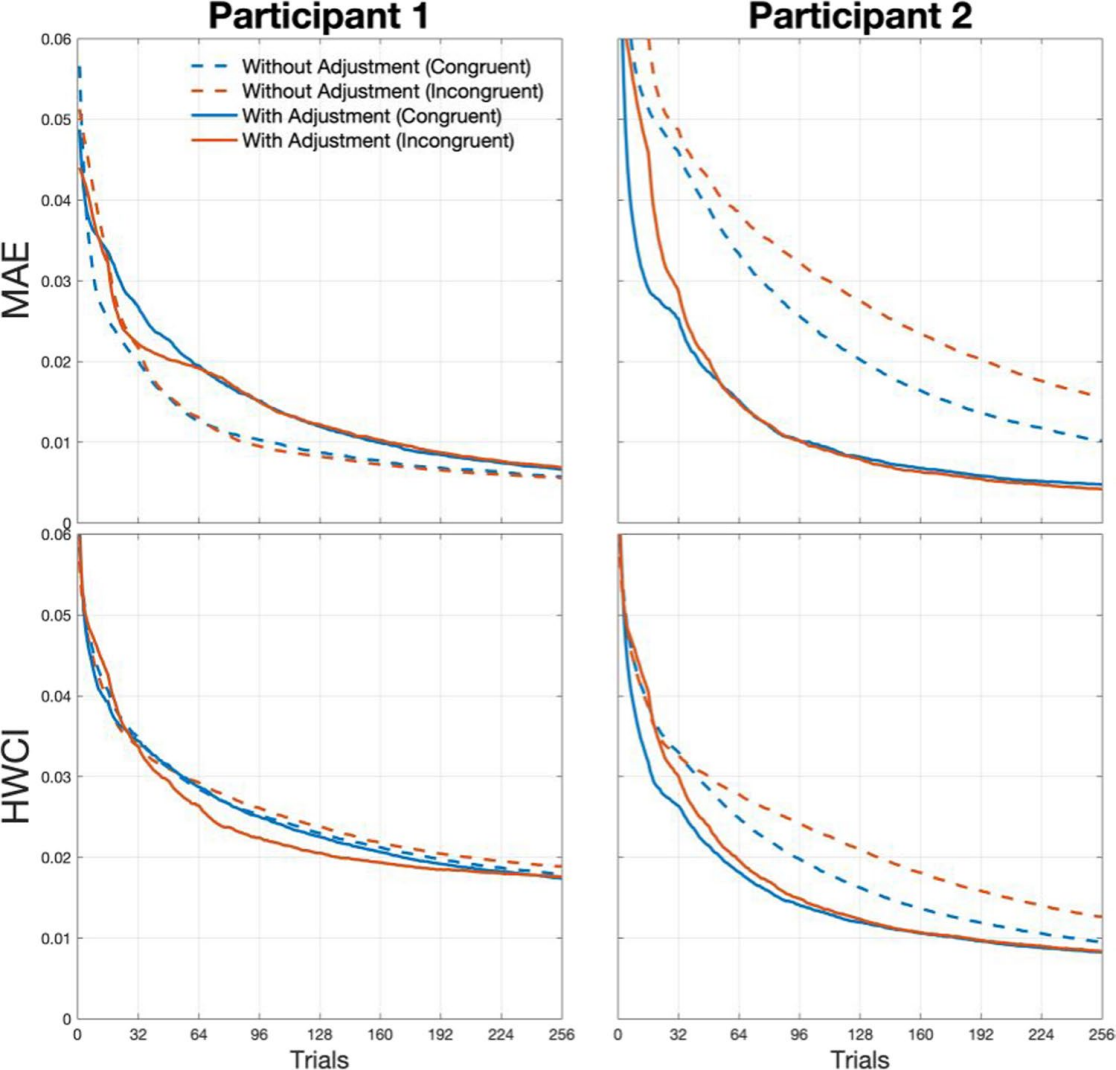
Accuracy (MAE; *upper panels*) and precision (HWCI; *lower panels*) of the SATf estimation without and with the adjustment of parameter space as a function of completed trial numbers. For participant 2, the method shows smaller MAE and HWCI with the adjustment than without adjustment

The efficiency of the method was also evaluated by examining the accuracy and precision of estimated SATf as a function of the number of simulated trials (Fig. 6). The adjustment of *λ* space did not significantly affect the accuracy and precision in estimating participant 1’s SATf. MAE was smaller for the method without the adjustment than with the adjustment at the beginning of the simulation. Still, the difference became smaller as trials iterated. Finally, the bias of methods with and without adjustment converged around 180th trials (the difference in MAE between the two methods was 0.0018 and 0.0025 in the congruent and incongruent conditions, respectively). To reach the accuracy of MAE = 0.01, the method required 92 and 83 trials for the congruent and incongruent conditions, respectively, without the adjustment, but 151 and 156 trials for the congruent and incongruent conditions, respectively, with the adjustment. The HWCIs of both methods were almost equivalent during all trials (the maximum difference between the two methods was 0.0027 at the first trial in the congruent and 0.0041 at the 78th trial in the incongruent condition). To reach the precision of HWCI = 0.02, the method required 175 and 191 trials for the congruent and incongruent conditions, respectively, without the adjustment, but 164 and 130 trials for the congruent and incongruent conditions, respectively, with the adjustment.

However, there was a significant difference in accuracy for estimating participant 2’s SATfs. The MAE was much greater without the adjustment than with the adjustment at the beginning and did not converge even after the 256th trial in both congruent and incongruent conditions. The difference between MAEs was 0.0054 and 0.0115 in the congruent and incongruent conditions, respectively, at the 256th trial. The method without the adjustment reached the accuracy of MAE = 0.01 after the 247th trial in the congruent condition but not until the end of the simulation (i.e., 256th trial at least) in the incongruent condition. On the contrary, it required only 94 and 92 trials in the congruent and incongruent conditions, respectively, for the same accuracy. The method also showed better precision with the adjustment than without the adjustment. It required 91 and 133 trials in the congruent and incongruent conditions, respectively, for the precision of HWCI = 0.02 without the adjustment, while it took 53 and 60 trials in the congruent and incongruent conditions, respectively, for the same precision with the adjustment.

In sum, accuracy was greatly influenced by the participant’s true *λ* value with the method without adjustment, while it was relatively stable regardless of the true *λ* value with the method with adjustment. These results implied that the method became more robust for estimating a SATf with extreme parameter value by adding extra padding on the parameter space.

## Psychophysical validation

**Fig. 7:**
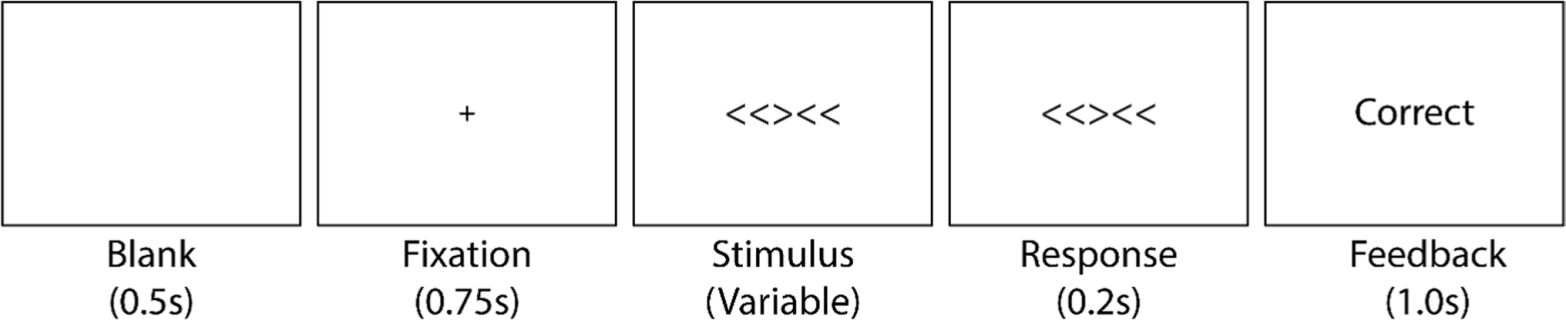
Illustration of the trial sequence in the psychophysical experiment. Each trial began with a fixation cross. The stimulus display contained five arrows and was followed by a response cue (beep sound). Participants were asked to report the direction of the central arrow. Feedback followed each response for 1 s

We evaluated the performance of the adaptive SAT method incorporating the stimulus selection algorithm based on the sum rule and the adjustment of *λ* space in a psychophysical experiment. Five young healthy participants ran a SAT version of the flanker task. They were asked to report the direction of the target arrow on the center while ignoring the other flanker arrows on the sides (see Fig. 7). The target arrow pointed in the same direction as flanker arrows in half of the trials (congruent) but in the opposite direction in the other half (incongruent). Half of the trial blocks were for MCS, and the others were for the adaptive SAT method. To evaluate the empirical validity of the adaptive SAT method, we compared SATfs obtained with the adaptive and the conventional SAT method (i.e., MCS).

## Methods

### Participants

Five naive participants participated in the flanker experiment. All participants were between 32 and 35 years and had corrected-to-normal vision. They had no experience in psychophysical studies. All participants gave written informed consent before participation. The Internal Review Board approved this study at Yonsei University Severance Hospital.

### Apparatus

The experiment was conducted on an IBM PC-compatible laptop computer. The stimuli were displayed on a 15.6-inch LCD monitor with a refresh rate of 60 Hz. The display was viewed at a distance of approximately 57 cm. The procedure—stimulus selection, presentation, and data collection—was controlled using MATLAB (The MathWorks, MA, USA) and Psychtoolbox extensions (Brainard, 1997; Pelli, 1997).

### Design

The SATfs were estimated with one MCS and four adaptive SAT runs for each participant in four sessions. Each session consisted of 512 adaptive trials and 512 MCS trials. For MCS, eight SOA blocks were repeated twice, and each block included 16 trials for each congruent and incongruent condition (512 MCS trials = 8 SOAs × 2 repetitions × 16 trials × 2 congruencies). The adaptive SAT measurement consisted of 16 blocks, each including 16 trials for each congruency (512 adaptive trials = 16 blocks × 16 trials × 2 congruencies). The estimation methods - MCS and the adaptive SAT - were interleaved across blocks. Two congruency trials were randomly interleaved in each block.

### Procedure

**Fig. 8:**
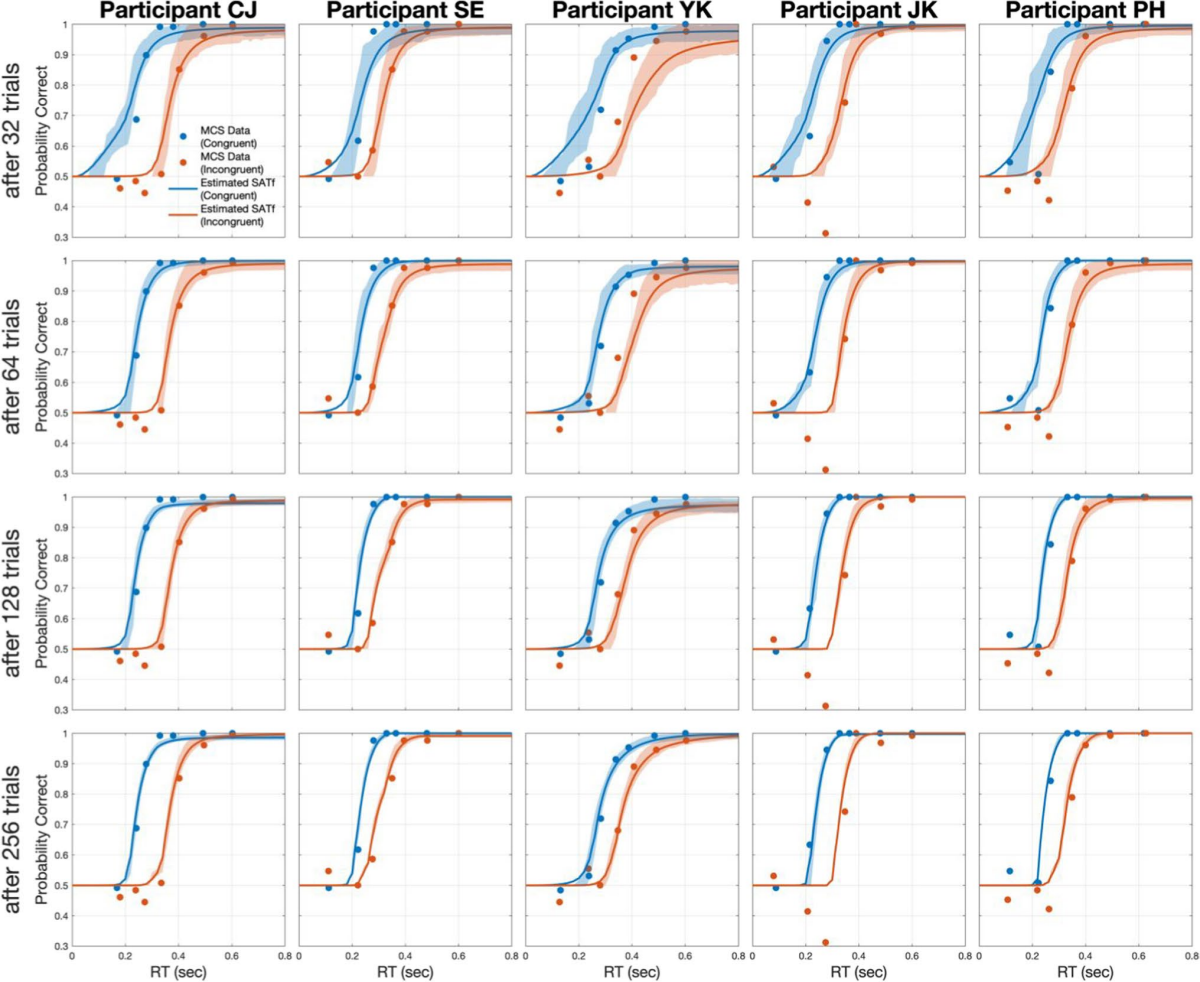
SATfs estimated by the adaptive SAT method (*lines*), along with *pc*s measured by MCS (*dots*), after a different number of adaptive trials. SATfs estimated with 32 adaptive trials exhibit large credible intervals and errors from *pc*s of 1024 MCS trials (*top panels*). However, errors and credible intervals quickly decrease afterward (*middle panels*). After the 256th trial, SATfs with the adaptive SAT method showed excellent agreement with MCS data and precision in all participants and conditions (*bottom panels*)

At the beginning of each trial, a fixation point was presented for 0.75 s at the center of the screen. Then a stimulus display containing five arrows was presented. The target (i.e., central arrow) was pointed either left or right (Fig. 8). In congruent trials, all the other four flanker arrows on the sides were directed the same direction as the target (e.g., <<<<< or >>>>>) in congruent trials, and the opposite direction of the target in incongruent trials (e.g., <<><< or >><>>). Each arrow subtended a visual angle of 1.83° × 1.83°. The response signal was then presented for 0.2 sec with a beep sound after a selected cue delay (SOA). SOA was selected from 0.06, 0.09, 0.12, 0.24, 0.36, 0.48, 0.60 and 1.20 s for each MCS block in a random order. For the adaptive SAT trials, the SOA level of each block was selected among 49 linearly spaced values between 0 and 1.2 s by the adaptive SAT method. Participants were asked to judge the direction of the central arrow while ignoring the other flanker arrows on the sides when the beep cue was sounding. A text of feedback (‘Correct’, ‘Incorrect’ or ‘Too late’) followed each response for 1 sec. Each session took approximately 1 h.

## Results

All responses of MCS trials were grouped into eight RT bins (from fastest to latest). Then, *pc* and mean RT were calculated for each RT bin. Agreement between *pc*s obtained with MCS and the adaptive method was evaluated by the root mean squared error (RMSE) between *pc*s estimated from the two methods at the eight bins in the MCS procedure:

15 $$RMSE=\sqrt{\frac{\sum_{j}\sum_{i}{\left({pc}_{ij}^{a}-{pc}_{i}^{MCS}\right)}^{2}}{I\times J}}$$

where ${pc}_{ijk}^{a}$ is *pc* at the *i*-th bin of the *j*-th run of the adaptive SAT method, and ${pc}_{i}^{MCS}$ is *pc* at the *i*-th SOA in the MCS trials.

There was a high concordance between SATfs estimated with the adaptive method and MCS (Fig. 8). Agreement between the two methods was evaluated by RMSE between *pc*s measured by MCS and SATf estimated by the adaptive SAT method. The RMSE, averaged by all participants and congruencies, was at 0.135 at the beginning (i.e., after the first adaptive trial). Then, it decreased to 0.082, 0.058, 0.050, and 0.047 at 32, 64, 128, and 256 trials, respectively. It was higher in the incongruent than the congruent condition (0.035 and 0.059 in the congruent and incongruent conditions, respectively, after the 256th trial). The higher RMSE in the incongruent condition might be due to fitting errors at the shorter SOA in some participants. We used a conventional SAT model where a SATf remains at the chance level at the shorter SOAs until the time point of *δ*, then begins to rise afterward. However, our MCS data shows a dip below the chance level just before *δ* in some participants—remarkably in participant JK. Though the dip increased overall RMSE between the two measurements, SATfs estimated with the adaptive method were matched to MCS data very well in other SOAs, condition, and participants.

Precision was greatly improved with the adaptive SAT trials as well. The HWCI of the estimated SATfs decreased with the trial number, starting from 0.062 after the first trial and reaching .026, .015, .011, and .006 at 32, 64, 128, and 256 adaptive SAT trials, respectively.

Results showed that the method well estimated SATfs in both congruent and incongruent conditions. In addition, the method properly estimated SATfs with a high asymptote. Since the flanker experiment is a relatively easy cognitive task, *pc* could be close to 100% if sufficient time is given for responses (i.e., long SOA). Indeed, participants’ SATfs saturated around 100% of *pc*, where the tendency of underestimation was observed in the original method. With the modified method, the underestimation was not noticeable in the experiment.

To measure the consistency of measurements, we evaluated the test–retest reliability of SATfs obtained with the adaptive SAT method. Results indicated that the adaptive SAT method could provide reliable estimates. The correlation between *pc*s measured in the first two and second two sessions was 0.991, and the slope of the linear regression between them was 1.020 (CI: 0.989–1.051, *r*^2^ = 0.982) at 256 adaptive SAT trials. In sum, results from the psychophysical study revealed that 128 adaptive trials could provide precise and accurate SATf estimation as much as 1024 MCS trials.

## Discussion

There is no doubt that hurried responses could make more errors. The tradeoff between speed and accuracy of responses, which has often been observed in psychophysical data and our daily life, obfuscates data interpretation. For an integrated account of RT and accuracy in various cognitive experiments, researchers have developed the SAT paradigm for data collection and the SAT model for data interpretation. Although the SAT paradigm and model could provide a fundamental characterization of the participant’s responses, it has not been the standard experimental paradigm in cognitive laboratories because it requires a long time for the assessment. To ease the SAT data collection and model estimation, we developed the original adaptive SAT method taking advantage of trial-by-trial Bayesian parameter estimation and the optimal stimulus searching for the maximum information (Baek & Park, 2021). By simulation, the adaptive method was proved to be highly efficient for SAT assessments: 50–150 trials were sufficient to achieve reasonable accuracy and precision levels. However, the original method has potential risks in its robustness and limited applicability to general task designs. Since it was designed to estimate a single SATf, it could suffer from inaccuracy and imprecision when assessing multiple SATfs in studies with a within-subject design. Moreover, it is prone to underestimating the upper asymptote in SATf when the task is easy, or the participant has a low lapse.

In the current study, we modified the original version of the adaptive SAT method to further enhance its robustness and applicability without sacrificing efficiency. By selecting a stimulus level (i.e., SOA) based on the sum of information gain from multiple experimental conditions (e.g., congruent and incongruent), the method could estimate all SATfs in multiple conditions accurately and precisely. By setting up extra padding in parameter space and truncating the final estimation at the theoretical limit, the underestimation could be corrected.

We validated the modified method with computer simulations and a psychophysical experiment of a flanker task. Simulation results showed that the sum-based stimulus selection strategy enabled the method to measure multiple SATfs without inaccuracy and imprecision in any condition. In addition, the extra padding in the parameter space ensured robust assessments of all potential participants (or tasks) while minimizing the effects of extreme values. Results from the psychophysical experiment confirmed the efficiency of the adaptive method. Estimates of the SATf obtained with 128 adaptive SAT trials were precise (HWCI = 0.011) and concordance with those obtained with 1024 MCS trials (RMSE = 0.050). Results suggested that the modifications allow the method to be successfully applied to more general study settings, such as a study containing multiple experimental conditions or relatively easy tasks.

The adaptive SAT method suggested in the current study generally showed good performance for estimating multiple SATfs simultaneously in most cases but showed limitations under certain extreme circumstances. As shown in simulation 1, the estimation was not precise for the incongruent condition in participant 3 due to the lack of information gain overlaps between the SATfs of the congruent condition and the incongruent condition with an exceptionally slowly increasing SATf without reaching the asymptote even at the longest SOA for general populations. Therefore, the current method is appropriate for the case when two SATfs overlap to a degree to estimate the SATfs of both conditions simultaneously.

Results from the psychological validation tell us a critical issue: a requirement of a suitable SATf model for a psychophysical test. The *pc*s measured with MCS included a dip below the chance level only for the incongruent trials in some participants. It indicates that some participants responded based on surrounding flankers or the entire stimulus array, not on the central target, at some shorter RTs. The early dip in the congruent condition has been reported in other SAT experiments under specific experimental designs, trial conditions, participant groups, and tasks (Gratton et al., 1988, 1992; Heitz & Engle, 2007; Stins et al., 2007). Thus, the errors are mainly attributable to the suboptimality of the SATf model to deal with the shorter SOA in the incongruent flanker task rather than the inefficiency of the current adaptive method. The present application to the flanker task is an example developed based on the standard model for SATf (Eq. 1), which did not take *pcs* below the chance level into account. For successful application of the adaptive estimation, it demands a more sophisticated SAT function for the flanker task, including the early dip. The adaptive scheme for multiple conditions proposed in the current study is expected to work for any SATf, including a more appropriate SATf model for the flanker task. However, the invention of the new SAT model for the flanker task is beyond the scope of this study, and we leave it as future research.

In a lengthy experiment, learning, habituation, or some errors due to fatigue and inattention might change the underlying psychological functions during the measurement. These could be compounding factors or sources of data noise but have often been ignored in data analysis if these are not the main interest of the study (e.g., learning studies). Thus, besides the convenience of data collection, it is the necessity of all quick estimation methods: taking a “snapshot” of the cognitive state before changes occur during an experiment. Even in the adaptive SAT procedure, some lengthy SOA may be confounding factors that affect the behaviors differently from those of the shorter SOA. This would be a critical issue in some complex tasks that need sustained mental processing. However, the current study's task is simple and does not require high mental processing for each trial unless SOA is too short.

We also consider that the short testing time of the adaptive SAT method could pose a weakness in practice. All Bayesian adaptive methods are often vulnerable to lapses (e.g., finger errors) at the beginning of an experiment (Kontsevich & Tyler, 1999). Since Bayesian adaptive methods generally gain more information about the psychometric function in earlier trials than later ones, lapses in the first few trials greatly influence the estimation performance. It would cause more complexity when the task requires a learning or adaptation period, particularly in the early stage. The effect of lapse trials was investigated in a study in which another Bayesian adaptive method was developed for the partial report paradigm (Baek et al., 2016). In the simulation, a participant made random responses in the first one, three, and five trials, but the method recovered from the participant's initial lapse and obtained sufficiently accurate and precise estimation—comparable to one without initial lapses—after 100–130 trials. As the current algorithm follows the previous one, the effect of lapse is expected to be recovered within the first few minutes. To overcome these factors, researchers may run a short practice block before the main experiment to minimize lapse trials and familiarize participants with the task and SAT paradigm.

Originally, a SATf was modeled in *d'* unit (e.g., McElree & Dosher, 1989; Reed, 1973, 1976; Wickelgren, 1977), which was useful for dissociating sensitivity and response bias (Macmillan & Creelman, 1991). However, the current method adopted a SATf in *pc* unit (Heitz, 2014; Samavatyan & Leth-Steensen, 2009) for several reasons. First, in a flanker task, responses are acquired in a forced choice manner, which has been known to have little or no response bias. Second, our implementation computed the Bayesian update in the probability domain (i.e., *pc*). Third, a small difference in behavioral performance could be exaggerated when expressed in *d'* at the extreme performance level (i.e., *pc* = 1.0) because of the nonlinear relationship between *pc* and *d'*. Thus, the method with the model in *d’* requires broad parameter space for λ, resulting in sampling inefficiency for parameter estimation. Last, the method needs to define a finite parameter space of *λ,* whereas *d’* does not have an upper limit by theory. Depending on the upper bound of *λ* in *d’* unit, there could be an underestimation or overestimation. For these reasons, SATf was modeled with *pc* unit in the adaptive SAT method.

In the current study, there are several methodological contributions. The first is to propose a method to simultaneously estimate the SATfs of two different conditions, the first in the Bayesian adaptive procedure field. So far, many have attempted to evaluate psychometric functions for processing a single cognitive condition; however, not many studies have dealt with multiple conditions. The current study's introduction of a method for estimating the SATfs of multiple conditions contributes not only to the Bayesian adaptive SATf estimation field but also to the psychometric estimation field. The second contribution we consider is the simple solution for parameter estimation of a restricted range via an appropriate prior. This technique can be a sort of problem-solving encountered in different situations. In estimating the SATf, however, the problem we solve is not specific to the current task but is fundamental in the Bayesian model parameter estimation of a psychometric function. Studies of SATf or other psychometric functions have not considered this issue sufficiently and thus have shown underestimation for an extremely high asymptote parameter (e.g., Baek et al., 2016). To resolve this and increase estimation precision, an experiment demands many samples, which is not a good choice for the adaptive estimation purpose. Instead, by solving this problem by extending the prior over the limited range, the current paper would contribute to many studies in the Bayesian parameter estimation with a restricted-range parameter. Finally, and mostly, the present study is significant in showing the practical validity of the proposed adaptive method compared to our previous study, which was primarily based on theoretical formulation.

Liu and Smith (2009) suggested a research procedure for estimating multiple SATfs using the best model selection among nested models from offline MCS-based data using MLE for parameter estimation. Here the “best” implies a model that can succinctly explain data from multiple conditions, considering the balance between the goodness of fit and the complexity of the model, with some parameters equal and others different across conditions. In contrast to their focus on model selection, our method primarily aims to estimate parameters (and functions) online and select stimulus levels adaptively for optimal SATf estimation with minimal trials. We could conduct model selection in the current Bayesian framework among the nested models from the model with all possible parameters. However, this will be only for offline processing, which is not appropriate for the online adaptive SATf estimation. For the online application, we may start from a model selected based on the task-specific hypothesis or previous experiment. Despite online, the final model parameters converge to the offline estimate parameters estimated using conventional model estimation techniques in the previous paper (Baek & Park, 2021) with a single condition and also converge to the ground truth in the current simulations with two conditions. Under the assumption that the full model is the best out of all candidate nested models, the adaptive SAT method would be more accurate and precise than the MLE approach for MCS data, especially with a limited number of trials, as reported in our previous study (Baek & Park, 2021).

Unfortunately, both approaches are vulnerable when two conditions are not based on the same functional form of the model (Eq. 1). For example, in the incongruent condition of JK’s dataset in our psychophysical validation, both approaches showed relatively poor goodness of fit compared to those in other participants or the congruent condition. The r^2^ was 0.913 between 1024 MCS trials and the SATf estimated by MLE with these data, and 0.895 between 1024 MCS trials and the SATf assessed by 256 adaptive trials.

Since the SAT phenomenon is almost ubiquitous in psychophysical studies, the adaptive SAT method would be helpful for studying characteristics of information processes and decision criteria when combined with virtually all experimental paradigms measuring RT and accuracy. Thus, it could be useful in many cognitive laboratories running psychophysical experiments for the average population. Furthermore, we believe it has great potential for studying cognitive characteristics in pathology. It has been reported that, for example, information processing speed was slower in patients with schizophrenia or Parkinson’s disease than in the healthy control group. In contrast, ADHD patients reported having the same information processing speed as the healthy control (Sergeant & Scholten, 1985a, b) but less flexible decision criteria (Mulder et al., 2010). The cognitive characteristics of those patients or other special populations (e.g., elderly people or young children) can be investigated by measuring SATfs, and the feasibility of the studies would be increased by a short testing time of the adaptive SAT method with improved robustness and applicability.

The efficiency of the Bayesian adaptive SAT method can be further improved by an informative prior (King-Smith et al., 1994). In our simulations and psychophysical validation, the prior distribution was uninformative at the beginning of the experiment (i.e., uniform distributions). In practice, researchers might set up a more informative prior using prior knowledge (e.g., pilot experiments or literature). It has been reported that a weakly informative prior improves the accuracy and precision of a Bayesian adaptive method if it is proper, although there exists a risk of deteriorated accuracy when the prior is informative but inappropriate (Baek et al., 2016; King-Smith et al., 1994). The prior can also be set using the hierarchical adaptive design optimization (HADO) (Gu et al., 2016; Kim et al., 2014; Zhao et al., 2021). The efficiency of the Bayesian adaptive estimation methods was proved to be enhanced more with the HADO framework. Therefore, it would be worth applying the prior distribution informed using prior knowledge or HADO framework to the current version of the adaptive SAT method.

The usability of the Bayesian adaptive SAT method should be improved for its popularity in clinical settings. In the current study, we implemented the method for the psychophysical study in laboratories. If it is implemented as a standalone application running on a desktop or a tablet computer, it would be more convenient for researchers to use it for data collection. Alternatively, the adaptive SAT method can be implemented in a gamified version, such as a mobile-friendly app. We hope such a development would increase the usability of the proposed method and help more researchers to collect higher-quality data from a range of populations.

## Data Availability

All datasets and codes for the experiment are available to download at a public repository (https://osf.io/wu9n6/).

## Appendix. Mathematical proof for the entropy of responses

$${H}_{b+1}\left({R}_{\widehat{x}}^{\left(1\right)},{R}_{\widehat{x}}^{\left(2\right)}\right)\triangleq \sum_{{r}_{\widehat{x}}^{\left(1\right)}\in \left\{\mathrm{0,1},\cdots ,{n}_{1}\right\}}\sum_{{r}_{\widehat{x}}^{\left(2\right)}\in \left\{\mathrm{0,1},\cdots ,{n}_{2}\right\}}{p}_{b+1}\left({r}_{\widehat{x}}^{\left(1\right)}\right){p}_{b+1}\left({r}_{\widehat{x}}^{\left(2\right)}\right)\left\{\mathrm{log}{p}_{b+1}\left({r}_{\widehat{x}}^{\left(1\right)}\right)+\mathrm{log}{p}_{b+1}\left({r}_{\widehat{x}}^{\left(2\right)}\right)\right\}=\sum_{{r}_{\widehat{x}}^{\left(2\right)}\in \left\{\mathrm{0,1},\cdots ,{n}_{2}\right\}}{p}_{b+1}\left({r}_{\widehat{x}}^{\left(2\right)}\right)\sum_{{r}_{\widehat{x}}^{\left(1\right)}\in \left\{\mathrm{0,1},\cdots ,{n}_{1}\right\}}{p}_{b+1}\left({r}_{\widehat{x}}^{\left(1\right)}\right)\mathrm{log}{p}_{b+1}\left({r}_{\widehat{x}}^{\left(1\right)}\right)+\sum_{{r}_{\widehat{x}}^{\left(1\right)}\in \left\{\mathrm{0,1},\cdots ,{n}_{1}\right\}}{p}_{b+1}\left({r}_{x}^{\left(1\right)}\right)\sum_{{r}_{\widehat{x}}^{\left(2\right)}\in \left\{\mathrm{0,1},\cdots ,{n}_{2}\right\}}{p}_{b+1}\left({r}_{\widehat{x}}^{\left(2\right)}\right)\mathrm{log}{p}_{b+1}\left({r}_{\widehat{x}}^{\left(2\right)}\right)=\sum_{{r}_{\widehat{x}}^{\left(2\right)}\in \left\{\mathrm{0,1},\cdots ,{n}_{2}\right\}}{p}_{b+1}\left({r}_{\widehat{x}}^{\left(2\right)}\right){H}_{b+1}\left({R}_{\widehat{x}}^{\left(1\right)}\right)+\sum_{{r}_{\widehat{x}}^{\left(1\right)}\in \left\{\mathrm{0,1},\cdots ,{n}_{1}\right\}}{p}_{b+1}\left({r}_{\widehat{x}}^{\left(1\right)}\right){H}_{b+1}\left({R}_{\widehat{x}}^{\left(2\right)}\right)={H}_{b+1}\left({R}_{\widehat{x}}^{\left(1\right)}\right)+{H}_{b+1}\left({R}_{\widehat{x}}^{\left(2\right)}\right)$$
